# Isolated intracranial hypertension associated with COVID-19

**DOI:** 10.1177/0333102420965963

**Published:** 2020-11-04

**Authors:** Marcus Tulius T Silva, Marco A Lima, Guilherme Torezani, Cristiane N Soares, Claudia Dantas, Carlos Otávio Brandão, Otávio Espíndola, Marilda M Siqueira, Abelardo QC Araujo

**Affiliations:** 1 Evandro Chagas National Institute of Infectious Diseases (INI), FIOCRUZ, Brazilian Ministry of Health, Brazil; 2 Neurology Department, Niteroi Hospital Complex, Niterói, Brazil; 3 Neurology Section, Clementino Fraga Filho University Hospital, UFRJ, Rio de Janeiro; 4 Neurology Department, Fluminense Federal University (U.F.F.), Niterói, Brazil; 5 Hospital Federal dos Servidores do Estado, Rio de Janeiro, Brazil; 6 Eye Hospital, Niterói, Brazil; 7 Neurolife Laboratory, Rio de Janeiro, Brazil; 8 Laboratory of Respiratory Virus and Measles of the Oswaldo Cruz Institute (IOC), FIOCRUZ, Brazilian Ministry of Health, Brazil; 9 Institute of Neurology, the Federal University of Rio de Janeiro (INDC-UFRJ), Brazil

**Keywords:** COVID-19, SARS-CoV-2, intracranial hypertension, pseudotumor cerebri, headache

## Abstract

**Background:**

Headache is a frequent complaint in COVID-19 patients. However, no detailed information on headache characteristics is provided in these reports. Our objective is to describe the characteristics of headache and the cerebrospinal fluid (CSF) profile in COVID-19 patients, highlighting the cases of isolated intracranial hypertension.

**Methods:**

In this cross-sectional study, we selected COVID-19 patients who underwent lumbar puncture due to neurological complaints from April to May 2020. We reviewed clinical, imaging, and laboratory data of patients with refractory headache in the absence of other encephalitic or meningitic features. CSF opening pressures higher than 250 mmH_2_O were considered elevated, and from 200 to 250 mmH_2_O equivocal.

**Results:**

Fifty-six COVID-19 patients underwent lumbar puncture for different neurological conditions. A new, persistent headache that prompted a CSF analysis was diagnosed in 13 (23.2%). The pain was throbbing, holocranial or bilateral in the majority of patients. All patients had normal CSF analysis and RT-qPCR for SARS-CoV-2 was negative in all samples. Opening pressure >200 mmH_2_O was present in 11 patients and, in six of these, > 250 mmH_2_O. 6/13 patients had complete improvement of the pain, five had partial improvement, and two were left with a daily persistent headache.

**Conclusions:**

In a significant proportion of COVID-19 patients, headache was associated to intracranial hypertension in the absence of meningitic or encephalitic features. Coagulopathy associated with COVID-19 could be an explanation, but further studies including post-mortem analysis of areas of production and CSF absorption (choroid plexuses and arachnoid granulations) are necessary to clarify this issue.

Several neurological manifestations associated with SARS-CoV-2 infection have been described since the beginning of the pandemic. In a cohort of hospitalized severe COVID-19 patients, neurological complaints occurred in 45.5% of infected individuals, varying from 1–14 days from the onset of SARS-CoV-2 infection (1). The most relevant were acute cerebrovascular disease in 5.7%, impaired consciousness in 14.8%, and skeletal muscle injury in 19.3%. In that clinical series, headache was observed in 28 out of 214 patients (13.1%).

According to the International Classification of Headache Disorders, headache attributed to systemic viral infection is diagnosed when there is an association between the onset of pain and systemic viral infection in the absence of encephalitic or meningitic features (2). The frequency of headaches in COVID-19 patients ranges from 5–34% according to different clinical series (3–8). However, no detailed information on headache characteristics is presented in these reports. Moreover, these clinical series do not describe cerebrospinal fluid (CSF) characteristics in these patients.

Herein, we describe the characteristics of headache and the CSF profile during SARS-CoV-2 infection in a consecutive series of COVID-19 patients, highlighting the cases associated with isolated intracranial hypertension. One of these cases is presented as an illustrative report.

## Methods

From April to May 2020, we retrospectively selected all consecutive inpatients with SARS-CoV2 infection from different centers who underwent lumbar puncture due to different neurological symptoms. We analysed those who presented refractory headaches with or without visual symptoms as the predominant neurological complaints and excluded those who presented any clinical or laboratory evidence for meningitis or meningoencephalitis, such as neck stiffness, altered consciousness, focal neurological signs, or inflammatory characteristics in the CSF analysis. For this study, we considered CSF opening pressures less than 200 mmH_2_O to be normal, higher than 250 mmH_2_O to be abnormal, and 200 to 250 mmH_2_O equivocal (9–11). All patients underwent lumbar puncture awake and in lateral decubitus. In all cases, SARS-CoV-2 RNA was detected by RT-qPCR through nasal and oropharyngeal swabs (Biomanguinhos kit (E+P1), FIOCRUZ, Brazil). The Local Ethical Committee at INI/FIOCRUZ approved this study.

## Results

### Case description

**Figure 1.**
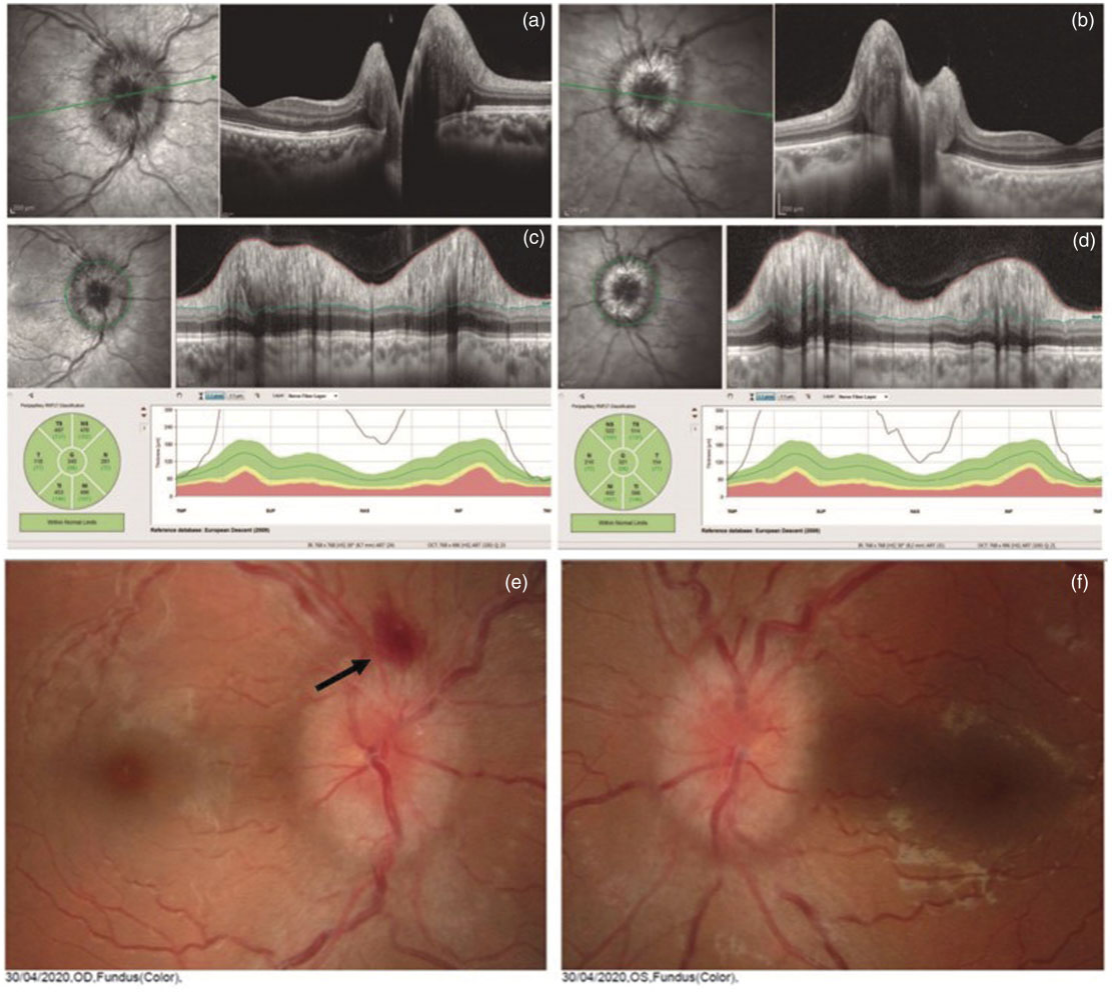
A 26-year-old female presenting bilateral papilledema related to post COVID-19 infection. The optic nerve head OCT B scans show elevation of the optic disc in both eyes ((a) and (b)). The peripapillary OCT circle scans show increased retinal nerve fiber layer thickness in all sectors ((c) and (d)). Retinography ((e) and (f)) showing papilledema and haemorrhage (arrow) in the right eye and papilledema in the left eye. In both eyes, oedema of the optic disc with high elevation is observed.

A 26-year old previously healthy, not obese female presented with severe, holocranial throbbing headache with nausea, dizziness, and significant visual blurring on the second day past flu-like symptoms due to SARS-CoV-2 infection. Ophthalmological examination revealed bilateral optic disc oedema. Optical coherence tomography was suggestive of optic disc oedema (Figure 1). No focal deficit, ataxia, or pupil light response abnormalities were found on the neurological examination. Brain and orbital MRI were normal except for discrete white matter lesions in the pons and the cerebral hemispheres suggestive of small vessel disease best observed on T2/FLAIR sequences. Brain venous MRI found no evidence of cerebral venous thrombosis. On the eighth day, she underwent lumbar puncture. CSF analysis revealed an opening pressure of 350 mmH_2_O, 2 cells/mm^3^, 15 mg/dL of protein level, and 58 mg/dL of glucose. RT-PCR for SARS-CoV-2 was negative in the CSF. Due to the pontine lesion, extensive laboratory exams were required to exclude demyelinating disease. CSF oligoclonal bands were not present, and serum anti-NMO (CBA method) was negative. No other causes of intracranial hypertension were found. The patient was treated with acetazolamide 500 mg bid and had partial improvement of symptoms to date.

The clinical and laboratory characteristics of this clinical series are presented in Table 1. In this study, 56 consecutive COVID-19 patients underwent lumbar puncture for different neurological conditions during the SARS-CoV-2 infection, and 13 of these (23.2%) had a new, persistent headache that prompted a CSF analysis. The most frequent complaint in these patients was a throbbing, holocranial, or bilateral intense daily headache. In four patients, there was a previous history of migraine without aura. However, the current headache was different, with more intense and more persistent pain than usual for them. Other relevant neurological complaints in these patients were peripheral facial palsy in one and blurred vision in three, with documented papilledema in two; all other patients had normal fundoscopy.

**Table 1:** . Clinical and laboratorial characteristics of headache associated with SARS-CoV-2 infection.

Age/gender	Main COVID symptoms/sigs	Headache onset^1^	Headache characteristics and Nx symptoms/signs	CSF analysis^2^	Follow-up
42/M	Anosmia, fever, myalgia	12 days	Intense, daily, throbbing, holocranialLasted 4 days	200/120/2/59/39	Recovered
47/F	Myalgia, anosmia, cough	2 days	Intense, throbbing, frontotemporal bilateral Lasted 15 days	220/100/1/58/25	Recovered
42/M	Myalgia, fever, diarrhea	2 days	Intense, pressing or tightening, bilateral. Lasted 8 days	600/300/1/59/26	Recovered
27/F	Sore throat, cough, anosmia, fever	1 day	Orbital, intense, throbbing, blurred vision^3^, bilateral papilledema	210/120/2/51/31	Partial recovery. Acetazolamide 1.5g/day
26/F*	Cough, myalgia, fever	2 days	Intense, throbbing, pulsatile tinnitus, vertigo, blurred vision^3^, papilledema, and attention deficit	350/180/2/51/15	Partial recovery. Acetazolamide 3 g/day + Furosemide 20 mg/day
34/F	Fever, myalgia	0 day	Intense, daily, throbbing, bilateral frontotemporal, insomnia	150/90/1/56/30	Partial recoveryDaily, mild headache
34/F	Headache, myalgia	0 day	Intense, throbbing, frontotemporal, insomnia	210/120/2/71/22	Partial recovery
39/F	Headache, low backpain, D-dimer 10.000 ng/ml	0 day	Intense, throbbing, holocranial, left facial palsy (3rd day), attention deficit; lasted 3 days	300/100/1/38/31	Recovered
47/F	Headache, anosmia, cough, fatigue, fever; thorax CT w/50% of ground glass involvement	0 day	Intense, occipital, throbbing	250/150/1/61/25	Recovered
45/F	Fever, headache, diarrhea, sweating, anosmia	0 day	Intense, fluctuating, bilateral, non-throbbing headache	200/120/2/59/31	Not recovered; daily fluctuating headache, although less severe
35/F	Low fever, tiredness, dry cough, anosmia, confusional state, drowsiness	0 day	Moderate, daily, non-throbbing headache, without other features.^4^ Lasted 7 days	400/300/2/54/39	Recovered
68/M	Runny nose, asthenia, fever, cough	10 days	Progressively intense, daily, non-throbbing, occipital slowly spreading to the whole skull; dizziness and nausea	200/110/4/43/45	Refractory, intense, daily for 50 days. Slowly improved and to date is recovered
44/F	Myalgia, fever, cough, mild dyspnea	12 days	Intense, daily, holocranial pressure, sometimes throbbing, blurred vision associated with nausea	280/180/1/63/27	Recovered

*This patient appears in case description in the section Results of this manuscript.

^1^Headache onset after COVID symptoms/signs.

^2^Cerebrospinal fluid analysis: Opening pressure mmH_2_O/closing pressure mmH_2_O/cell count/mm^3^/glucose level mg/dl/protein level mg/dl.

^3^Transitory symptom, normal visual acuity test.

^4^Brain MRI unremarkable except for signs of intracranial hypertension (distension of perioptic subarachnoid space and empty sella).

The opening CSF pressure of one of these patients with papilledema was 210 mmH_2_O, below the level required for the diagnosis of pseudotumor cerebri syndrome (PTCS), according to a revised diagnostic criteria (12). All patients had normal cell counts and glucose and protein levels on the CSF analysis. Besides, RT-qPCR for SARS-CoV-2 in CSF was negative in all patients. Median values for cell counts, glucose, and protein were of 1.6/mm^3^ (1-4/mm^3^), 55.6 mg/dL (38–71mg/dL), and 29.6 mg/dL (15–45mg/dL), respectively. CSF opening pressure ranged from 150 to 600 mmH_2_O (median of 270 mmH_2_O). In 12 patients, the opening pressure was ≥200 mmH_2_O (median of 251 mmH_2_O) and in six of these ≥250 mmH_2_O (median of 355 mmH_2_O). No other causes for intracranial hypertension, such as obesity, venous thrombosis, or drugs, were identified in these patients. All patients were submitted to brain MRI and only in one patient were abnormalities typically seen in intracranial hypertension detected (see Table 1).

To date, nine out of 13 patients were pain-free 8 days after the headache onset on average (3–15 days), and five had a partial recovery, with mild headache (follow-up of 66 days to date).

Other neurological conditions that prompted lumbar puncture were meningoencephalitis/encephalopathy in 35 patients, Guillain-Barré syndrome in four, Miller-Fisher syndrome in two, and acute myelitis in two individuals. Median CSF opening pressure in this group was of 200 mmH_2_O (150–400).

## Discussion

In this clinical series, 13 patients had severe and persistent headache in the course of SARS-CoV-2 infection, which justified an analysis of the CSF. Of note, intracranial hypertension in the absence of meningitis/encephalitis was seen in 84.6% of patients (11 out of 13) assuming a normal CSF opening pressure of up to 200 mmH_2_O. If we consider the cut-off of 250 mmH_2_O, as proposed by the revised criteria for PTCS (12), 46.1% of our patients (six out of 13) still fulfil the criteria for intracranial hypertension.

Interesting, in our series, 10 out of 13 patients were women (Table 1). The prevalence of idiopathic intracranial hypertension is characteristically higher among women, at a rate of 3.3 per 100,000 per year in females aged 15–44 years, corresponding to a female: male ratio of 8:1 (13). We could consider that the same would be expected in cases of intracranial hypertension associated with COVID-19.

Recently, one case of benign intracranial hypertension was published (14). In this case report, a 35-year-old female patient presented headache 2 days before COVID-19 symptoms and signs, rapidly evolving to mental confusion. CSF analysis was normal except for a high opening pressure (400 mmH_2_O) and brain MRI disclosed prominent subarachnoid space around optic nerves, vertical tortuosity of the optic nerves, and superior compression of the hypophysis.

The normal value of CSF pressure in an appropriate clinical setting could be a matter of debate, with some arguing that pressures between 200 to 250 mmH_2_O are equivocal (15). Because the CSF pressure naturally fluctuates, intracranial pressure monitoring at another time may be necessary to establish the diagnosis with certainty. Patients with a transient or intermittent elevation of the CSF pressure below the threshold required to produce oedema at the optic nerve head are described. This could justify the absence of ophthalmological symptoms and signs (16). Furthermore, a classic example of acute neurological disease with elevated intracranial hypertension and the absence of papilledema in a significant proportion of cases is subarachnoid haemorrhage. In addition, patients with headache and elevated opening pressure on lumbar puncture but no papilledema have been described (17–21).

Only two of our patients had a documented papilledema. Although fundus was examined as part of neurological examination in all patients, not all enrolled patients were formally evaluated by an ophthalmologist, which is a limitation of our study. Optic fundi examination is part of neurological examination and of utmost importance in recognition of raised intracranial pressure. However, the protective additional measures such as face shield and close contact with patients during the exam impose additional difficulties to physicians evaluating COVID-19 patients with headache.

Importantly, patients without papilledema are at lower risk of vision loss compared to typical patients with intracranial hypertension with papilledema (12,17,22). Ophthalmologic evaluation is advised to confirm the presence of papilledema and to evaluate those patients with questionable or subtle papilledema (23).

Intracranial hypertension has been associated with diverse conditions such as obesity, autoimmune diseases, use of certain medications, vitamin deficiencies, vitamin excesses, bacterial infections, and viral infections such as HIV, measles, varicella, herpes virus, and hepatitis A virus (24–26).

The real pathogenesis of intracranial hypertension in the absence of an occupying mass lesion is a matter of debate. The main hypotheses indicate that it can be caused by increased abdominal and/or intracranial venous pressure, venous outflow abnormalities, alterations in absorption or production of CSF, low-grade inflammation, intracranial vascular clotting, or cerebral edema (15).

Recently, it has been described that SARS-CoV-2 infection can be associated with coagulation dysfunction, predisposing infected individuals to venous thromboembolism in several ways (27). Pathogenic mechanisms, including endothelial dysfunction with increased levels of von Willebrand factor, systemic inflammation with Toll-like receptor activation, and a procoagulatory state via tissue factor pathway activation, are involved (28). Intracranial venous thrombosis has been implicated as a cause for intracranial hypertension secondary to CSF outflow obstruction (29). Thus, we could speculate that venous congestion could be precipitated during a hypercoagulable state caused by SARS-CoV-2 infection.

Although our cases presented normal brain MRI, with magnetic resonance venography indicating normal venous sinuses, a possible hyperviscosity mechanism caused by SARS-CoV-2 would increase the venous pressure without actual venous sinus thrombosis. Thus, a low-grade inflammation determined by SARS-CoV-2 infection in conjunction with this hyperviscosity and hypercoagulable state could result in intracranial hypertension in some infected individuals. Indeed, in a recent study by Duarte-Neto et al., evidence of brain small vessel disease was observed in 30% of autopsies from COVID-19 patients (30). Further pathological studies are necessary to confirm this hypothesis.

A practical issue is that headaches secondary to intracranial hypertension occurred in a significant proportion of our patients, highlighting the importance of prompt recognition. Optic fundi examination is part of the neurological examination and of utmost importance in recognition of raised intracranial pressure. However, the additional protective measures such as the use of a face shield and the close contact with patients during this exam impose additional difficulties to physicians evaluating COVID-19 patients with headache. Additionally, an optic disc photo is an option as a diagnostic tool, but is only available in eye departments.

In our series, neurological complaints other than headache were peripheral facial palsy in one and cognition symptoms in two individuals, who reported poor concentration and inattention affecting some activities of daily living. Patients with intracranial hypertension do not usually have cognitive deficits. However, one study showed deficits in reaction time and processing speed that persisted on retesting at 3 months despite improvement in measured ICP and headache (31). Regarding peripheral facial palsy, it is infrequently reported in idiopathic intracranial hypertension (32,33). In addition, the involvement of the facial nerve has been associated with SARS-CoV-2 infection, but generally in the context of Guillain-Barré syndrome (34,35).

## Conclusion

Headache is one of the frequent neurological symptoms associated with COVID-19. According to the present study, in the absence of evidence of meningitis or cerebrovascular disease, headache can be severe, persistent, and associated to intracranial hypertension in a significant proportion of cases. The recently described coagulopathy associated with COVID-19 could be an explanation for such cases that could be due to the induction of a hyperviscosity state, leading to less absorption of CSF. Necropsy studies examining areas of production and CSF absorption; that is, choroid plexuses and arachnoid granulations, may help to clarify this issue.

## Clinical implications

What are the characteristics of headache in COVID-19 patients? What is the cerebrospinal fluid (CSF) profile of these individuals? In this cross-sectional study, 13 out of 56 COVID-19 patients submitted to CSF analysis had severe, persistent headache. In 11 patients, the opening pressure was above 200 mmH_2_O and in 6 of these, above 250 mmH_2_O. RT-qPCR for SARS-CoV-2 was negative in all samples. Headache can be attributed to isolated intracranial hypertension in the absence of meningitic or encephalitic features in a significant number of COVID-19 patients.

## References

[1] Mao L Jin H Wang M, et al Neurologic manifestations of hospitalized patients with coronavirus disease 2019 in Wuhan, China. JAMA Neurol. 2020; 77: 683–690. 32275288 10.1001/jamaneurol.2020.1127 PMC7149362

[2] Headache Classification Committee of the International Headache Society (IHS). The International Classification of Headache Disorders, 3rd edition. Cephalalgia 2018; 38: 1–211. 10.1177/0333102417738202 29368949

[3] Guan W-J Ni Z-Y Hu Y, et al Clinical characteristics of coronavirus disease 2019 in China. N Engl J Med. 2020; 382: 1708–1720. 32109013 10.1056/NEJMoa2002032 PMC7092819

[4] Chen N Zhou M Dong X, et al Epidemiological and clinical characteristics of 99 cases of 2019 novel coronavirus pneumonia in Wuhan, China: A descriptive study. Lancet 2020; 395: 507–513. 32007143 10.1016/S0140-6736(20)30211-7 PMC7135076

[5] Shi H Han X Jiang N, et al Radiological findings from 81 patients with COVID-19 pneumonia in Wuhan, China: A descriptive study. Lancet Infect Dis 2020; 20: 425–434. 32105637 10.1016/S1473-3099(20)30086-4 PMC7159053

[6] Huang C Wang Y Li X, et al Clinical features of patients infected with 2019 novel coronavirus in Wuhan, China. Lancet 2020; 395: 497–506. 31986264 10.1016/S0140-6736(20)30183-5 PMC7159299

[7] Yang X Yu Y Xu J, et al Clinical course and outcomes of critically ill patients with SARS-CoV-2 pneumonia in Wuhan, China: A single-centered, retrospective, observational study. Lancet Respir Med 2020; 8: 475–481. 32105632 10.1016/S2213-2600(20)30079-5 PMC7102538

[8] Xu X-W Wu X-X Jiang X-G, et al Clinical findings in a group of patients infected with the 2019 novel coronavirus (SARS-Cov-2) outside of Wuhan, China: Retrospective case series. BMJ 2020; 368: m606. 32075786 10.1136/bmj.m606 PMC7224340

[9] Friedman DI Jacobson DM. Diagnostic criteria for idiopathic intracranial hypertension. Neurology 2002; 59: 1492–1495. 12455560 10.1212/01.wnl.0000029570.69134.1b

[10] Corbett JJ Mehta MP. Cerebrospinal fluid pressure in normal obese subjects and patients with pseudotumor cerebri. Neurology 1983; 33: 1386–1388. 6684240 10.1212/wnl.33.10.1386

[11] Friedman DI. Papilledema and pseudotumor cerebri. Ophthalmol Clin North Am 2001; 14: 129–147, ix. 11370563

[12] Friedman DI Liu GT Digre KB. Revised diagnostic criteria for the pseudotumor cerebri syndrome in adults and children. Neurology 2013; 81: 1159–1165. 23966248 10.1212/WNL.0b013e3182a55f17

[13] Radhakrishnan K Ahlskog JE Cross SA, et al Idiopathic intracranial hypertension (pseudotumor cerebri). Descriptive epidemiology in Rochester, Minn, 1976 to 1990. Arch Neurol 1993; 50: 78–80. 8418804 10.1001/archneur.1993.00540010072020

[14] Noro F Cardoso F de M Marchiori E. COVID-19 and benign intracranial hypertension: A case report. Rev Soc Bras Med Trop 2020; 53: e20200325. 32520086 10.1590/0037-8682-0325-2020 PMC7294953

[15] Digre KB Corbett JJ. Idiopathic intracranial hypertension (pseudotumor cerebri): A reappraisal. Neurologist 2001; 7: 2–67.

[16] Spence JD Amacher AL Willis NR. Benign intracranial hypertension without papilledema: Role of 24-hour cerebrospinal fluid pressure monitoring in diagnosis and management. Neurosurgery 1980; 7: 326–336. 7442975 10.1227/00006123-198010000-00004

[17] Mathew NT Ravishankar K Sanin LC. Coexistence of migraine and idiopathic intracranial hypertension without papilledema. Neurology 1996; 46: 1226–1230. 8628457 10.1212/wnl.46.5.1226

[18] Wang SJ Silberstein SD Patterson S, et al Idiopathic intracranial hypertension without papilledema: A case-control study in a headache center. Neurology 1998; 51: 245–249. 9674810 10.1212/wnl.51.1.245

[19] Quattrone A Bono F Fera F, et al Isolated unilateral abducens palsy in idiopathic intracranial hypertension without papilledema. Eur J Neurol 2006; 13: 670–671. 16796599 10.1111/j.1468-1331.2006.01279.x

[20] Marcelis J and Silberstein SD. Idiopathic intracranial hypertension without papilledema. Arch Neurol 1991; 48: 392–399. 2012512 10.1001/archneur.1991.00530160060014

[21] Vieira DSS Masruha MR Gonçalves AL, et al Idiopathic intracranial hypertension with and without papilloedema in a consecutive series of patients with chronic migraine. Cephalalgia 2008; 28: 609–613. 18384415 10.1111/j.1468-2982.2008.01564.x

[22] Soler D Cox T Bullock P, et al Diagnosis and management of benign intracranial hypertension. Arch Dis Child 1998; 78: 89–94. 9534686 10.1136/adc.78.1.89 PMC1717437

[23] Krishnakumar D Pickard JD Czosnyka Z, et al Idiopathic intracranial hypertension in childhood: Pitfalls in diagnosis. Dev Med Child Neurol 2014; 56: 749–755. 24854011 10.1111/dmcn.12475

[24] Prevett MC Plant GT. Intracranial hypertension and HIV associated meningoradiculitis. J Neurol Neurosurg Psychiatry 1997; 62: 407–409. 9120463 10.1136/jnnp.62.4.407 PMC1074106

[25] Ravid S Shachor-Meyouhas Y Shahar E, et al Viral-induced intracranial hypertension mimicking pseudotumor cerebri. Pediatr Neurol 2013; 49: 191–194. 23831246 10.1016/j.pediatrneurol.2013.03.007

[26] Gilad O Shefer-Averbuch N Garty BZ. Primary varicella infection presenting with headache and elevated intracranial pressure. J Child Neurol 2015; 30: 793–795. 24846901 10.1177/0883073814535500

[27] Gris J-C Perez-Martin A Quéré I, et al COVID-19 associated coagulopathy: The crowning glory of thrombo-inflammation concept. Anaesth Crit Care Pain Med 2020; 39: 381–382. 32418867 10.1016/j.accpm.2020.04.013 PMC7196534

[28] Wichmann D Sperhake J-P Lütgehetmann M, et al Autopsy findings and venous thromboembolism in patients with COVID-19. Ann Intern Med 2020; 173: 268–277. 32374815 10.7326/M20-2003 PMC7240772

[29] Tamer SK Tamer U Warey P. Infantile pseudotumor cerebri related to viral illness. Indian J Pediatr 1996; 63: 645–649. 10830033 10.1007/BF02730810

[30] Dolhnikoff M Duarte-Neto AN de Almeida Monteiro RA, et al Pathological evidence of pulmonary thrombotic phenomena in severe COVID-19. J Thromb Haemost 2020; 18: 1517–1519. 32294295 10.1111/jth.14844 PMC7262093

[31] Yri HM Fagerlund B Forchhammer HB, et al Cognitive function in idiopathic intracranial hypertension: A prospective case-control study. BMJ Open 2014; 4: e004376. 10.1136/bmjopen-2013-004376 PMC3987738 24713214

[32] Capobianco DJ Brazis PW Cheshire WP. Idiopathic intracranial hypertension and seventh nerve palsy. Headache 1997; 37: 286–288. 9195767 10.1046/j.1526-4610.1997.3705286.x

[33] Selky AK Dobyns WB Yee RD. Idiopathic intracranial hypertension and facial diplegia. Neurology 1994; 44: 357. 8198627 10.1212/wnl.44.2.357

[34] Juliao Caamaño DS and Alonso Beato R. Facial diplegia, a possible atypical variant of Guillain-Barré Syndrome as a rare neurological complication of SARS-CoV-2. J Clin Neurosci 2020; 77: 230–232. 32410788 10.1016/j.jocn.2020.05.016 PMC7221378

[35] Ottaviani D Boso F Tranquillini E, et al Early Guillain-Barré syndrome in coronavirus disease 2019 (COVID-19): A case report from an Italian COVID-hospital. Neurol Sci 2020; 41: 1351–1354. 32399950 10.1007/s10072-020-04449-8 PMC7216127

